# Plant-Based Care and Therapy in Ophthalmology

**DOI:** 10.3390/antiox14121510

**Published:** 2025-12-16

**Authors:** Olga Klaudia Szewczyk-Roszczenko, Marta Pietruszyńska, Iga Anna Iwańska, Piotr Roszczenko, Krzysztof Bielawski, Agnieszka Gornowicz, Anna Bielawska

**Affiliations:** 1 Department of Synthesis and Technology of Drugs, Medical University of Bialystok, Kilinskiego 1, 15-089 Bialystok, Poland; krzysztof.bielawski@umb.edu.pl; 2 Department of Clinical Pharmacology, Medical University of Bialystok, Kilinskiego 1, 15-089 Bialystok, Poland; marta@pietruszynska.pl; 3 Department of Biotechnology, Medical University of Białystok, Kilinskiego 1, 15-089 Bialystok, Poland; igaannaiwanska@gmail.com (I.A.I.); piotr.roszczenko@sd.umb.edu.pl (P.R.); agnieszka.gornowicz@umb.edu.pl (A.G.)

**Keywords:** cataract, dry eye syndrome, macular degeneration, glaucoma, diabetic retinopathy

## Abstract

Oxidative stress, inflammation, and environmental factors contribute significantly to the development of ocular disorders, including dry eye disease, conjunctivitis, and age-related degenerative changes. In recent years, growing attention has been directed toward natural compounds and plant-derived extracts with potential protective and therapeutic effects on eye health. This work provides an overview of selected bioactive substances, such as carotenoids (β-carotene), flavonoids, vitamins C and E, and phytochemicals derived from plants. These agents exhibit antioxidative, anti-inflammatory, antimicrobial, and regenerative properties that may support ocular surface integrity, reduce oxidative damage, and improve visual performance. The integration of such natural remedies into ocular health strategies may offer complementary benefits to conventional therapies.

## 1. Introduction

Natural plant extracts have played a pivotal role in the field of medicine for millennia, with their utilization in the treatment of ocular diseases being a domain of mounting interest in both traditional and contemporary medicine. Medicinal plants have been shown to provide a wide range of bioactive compounds, including polyphenols, alkaloids, flavonoids, carotenoids, fatty acids, and antioxidants [1]. These bioactive compounds have the potential to promote tissue regeneration, reduce inflammation, improve microcirculation, and protect cells from oxidative stress. Modern science is increasingly confirming the effectiveness of natural substances in the prevention and treatment of various ailments, including eye diseases, which are increasingly common due to an aging population, exposure to environmental pollutants, and prolonged exposure to artificial light and electronic device screens [2].

Eye diseases such as macular degeneration, cataracts, glaucoma, diabetic retinopathy, and dry eye syndrome represent some of the most prevalent ophthalmological problems worldwide, and their treatment often necessitates long-term drug therapy or surgical intervention [3]. Consequently, natural plant extracts present an intriguing alternative or complement to conventional treatments, as they have the capacity to act both prophylactically and therapeutically, thereby promoting eye health. Lutein and zeaxanthin, compounds present in leafy vegetables and fruits, accumulate in the retina and filter out harmful blue radiation, thereby reducing the risk of macular degeneration [4]. Anthocyanins, found in blueberries and chokeberries, have been shown to enhance the elasticity of the eye’s blood vessels, which may contribute to the management of diabetic retinopathy and night vision impairment [5]. Polyphenols, present in green tea and red wine, act as potent antioxidants, protecting retinal cells from degeneration and free radical damage [6].

Beyond their role in ocular health, natural substances have also been shown to support nervous system function, a critical aspect in the management of neurodegenerative diseases such as glaucoma and optic neuropathy. Saffron, a spice derived from the saffron crocus, has demonstrated neuroprotective properties and may support the treatment of macular degeneration by enhancing the sensitivity of the retina to light stimuli [7]. Amla, a rich source of vitamin C, has been shown to strengthen blood vessels in the eye and reduce the risk of retinal hemorrhages [8]. Nigella seed oil has also shown protective effects against oxidative stress-related damage to the optic nerve [9].

Considering the rising prevalence of ocular diseases and the demand for effective yet natural and safe treatments, plant extracts present a promising solution that can be used for both prevention and therapy. Further scientific research in this field is recommended to facilitate a more profound comprehension of the mechanisms of action of individual plant substances and their potential application in modern ophthalmology.

## 2. Inflammatory and Infectious Diseases of the Eye

Inflammatory eye disease refers to a broad spectrum of conditions, ranging from relatively mild to potentially sight-threatening disorders. Ocular inflammation can arise from either infectious or non-infectious causes, and it may affect any part of the eye, including the conjunctiva, cornea, uvea, retina, and optic nerve. Inflammation may occur in isolation or as a manifestation of a broader systemic inflammatory disease [10].

The underlying causes of these conditions are diverse and may include bacterial, viral, fungal, or parasitic infections, as well as autoimmune mechanisms, trauma, or exposure to toxic agents. Accurate diagnosis and timely management are crucial, as untreated inflammation can lead to irreversible vision loss or other ocular complications.

### 2.1. Tea Tree Oil

Blepharitis and Meibomian Dysfunction (MGD) are chronic inflammatory conditions that affect the eyelid margins and the meibomian glands, respectively. They have a complex pathogenesis and can lead to tear film instability, eye irritation, and ocular surface disease [11]. The main chronic symptoms include redness, flaking, itching, crusting of the eyelids, a sensation of heavy eyelids, and blurry vision.

Among the potential etiological factors, particular attention has been given to *Demodex*, a mite that is a natural component of skin microbiota. While it is asymptomatic under normal conditions, excessive proliferation within eyelid structures can trigger inflammatory responses. The pathogenic role of *Demodex* in blepharitis has been confirmed in numerous studies [12].

A widely used treatment for *Demodex*-related blepharitis is tea tree oil (TTO). This is a natural oil distilled from the leaves of *Melaleuca alternifolia*, known for its antibacterial, antifungal, antiviral, antiprotozoal, and anti-inflammatory properties. It has proven effective against *Staphylococcus aureus*, *Pseudomonas aeruginosa*, *Escherichia coli*, and *Candida albicans*, and it is especially noted for its strong efficacy in eradicating *Demodex* [13]. In a study by Salva et al., patients treated with TTO for *Demodex* blepharitis showed a significant reduction in mite count, as well as an improvement in the severity of clinical symptoms [14]. Similarly, Koo et al. [15] investigated 335 patients with ocular *Demodex* infestation. The patients were randomly divided into two groups: 106 women received eyelid scrubs with TTO for one month, and their results were compared with 54 patients who did not receive TTO treatment. *Demodex* was found in 84% of patients experiencing ocular discomfort. The number of mites showed a significant positive correlation with Ocular Surface Disease Index (OSDI) scores and patient age. Based on their findings, TTO eyelid scrubs were found to be effective in eliminating ocular *Demodex* [15]. In clinical practice, ophthalmologists often recommend commercially available eyelid hygiene wipes soaked in appropriately concentrated tea tree oil. TTO has become a standard therapeutic option for the treatment of *Demodex*-related blepharitis.

### 2.2. Chamomile (Matricaria chamomilla)

*Matricaria chamomilla* is a widely recognized medicinal herb belonging to the *Asteraceae* family. This annual plant thrives under various soil conditions and exhibits strong resistance to cold temperatures. The plant contains apigenin, which has been shown to reduce inflammation and promote healing [16].

The study by Farsi et al. [17] utilized liquid cultures of *Staphylococcus aureus* and *Pseudomonas aeruginosa* to evaluate the efficacy of the containing chamomile extract. The eardrops were administered at increasing concentrations, while *S. aureus* and *Streptococcus pneumoniae* were tested with chamomile-containing eye drops at various time intervals (5, 10, 15, and 45 min). Bacterial viability was assessed using colony-forming unit (CFU) counts after an 18 h incubation and confirmed through resazurin microdilution assays. The results demonstrated that *P. aeruginosa* exhibited a reduction in CFUs within 5 min of exposure to any concentration of chamomile eardrops, with the greatest effect observed at a 30% concentration after 45 min. In the case of *S. aureus*, a similar trend was observed, with a substantial CFU reduction occurring within 5 min at all concentrations. In the experiments involving eye drops, a similar trend was observed, with a rapid decrease in CFUs, particularly at the 10% concentration, where the effect was almost immediate. *S. pneumoniae* also demonstrated a reduction in bacterial count as early as 5 min, with the effect persisting through the entire 45 min observation period at all tested concentrations [17].

### 2.3. Coptis teeta

*Coptis teeta* is a native Asia plant from the *Ranunculaceae* family. Recent studies have proven that it contains variety of alkaloids, such as berberine, palmatine, jatrorrhizine, coptisine, columbamine, and epiberberine. These compounds demonstrate anti-inflammatory, antimicrobial, and antioxidant properties. Unfortunately, due to its low reproductive success rate, it is considered to be an endangered species [18]. In ophthalmology it could be used more effectively than sulfacetamide to eradicate *Chlamydia trachomatis* and prevent symptoms relapse [19].

### 2.4. Aloe barbadensis Mill.

*Aloe barbadensis* Mill., usually called *Aloe vera*, is a tropical plant that belongs to the *Liliaceae* family and is characterized by long and juicy leaves that have a lot of pharmacologic properties, such as antimicrobial, anti-inflammatory, and antioxidant. These activities are due to various chemical compounds present in the previously mentioned leaves’ juice. These components are flavonoids, anthraquinones, phenolic acids, enzymes, and vitamins. To reduce inflammation, *Aloe vera* eye drops are used. Ethanolic and ethyl acetate are probably the most advantageous ingredients of *Aloe vera* in this case [20].

### 2.5. Quercetin

Flavonoids are abundant natural antioxidants found in a wide range of foods, including green leafy vegetables, tea, berries, apples, and onions [21]. Among them, quercetin, a prominent member of the flavonol subclass, has attracted considerable scientific interest in recent years. Like other flavonoids, quercetin acts as a potent antioxidant, capable of scavenging free radicals, chelating transition metal ions, and inhibiting lipid peroxidation [22,23].

Quercetin has been shown to exert strong antioxidant, anti-inflammatory, and anti-fibrotic effects both in vitro (Raw 264.7, A549 and BEAS-2B cells) and in vivo (BALB/c mice, CCl_4_-induced hepatic fibrosis in mice and rats) across various tissues. Its potential as a therapeutic agent has recently garnered attention within the field of ocular surface research, particularly in the study of dry eye disease, keratoconus, ocular surface inflammation, and corneal neovascularization [24]. Diseases affecting the ocular surface, such as those involving the cornea or conjunctiva, can lead to thinning, inflammation, scarring, and neovascularization of the cornea.

Multiple studies have emphasized the anti-inflammatory properties of quercetin, primarily through downregulation of the NF-κB signaling pathway, demonstrated both in vitro (murine small intestinal epithelial cell line, human mast cell line, HMC-1) [25,26] and in vivo (TNFDeltaARE/WT mice, peripheral blood human leukocytes) [27,28]. Additionally, quercetin has shown immunomodulatory effects when applied topically to the ocular surface in experimental models of dry eye [24]. In one such study, Oh et al. [29] administered 0.5% quercetin eye drops in a dry eye mouse model, resulting in a significant increase in tear volume and restoration of corneal surface smoothness, without damage to the corneal epithelium [29].

Furthermore, McKay et al. [30] demonstrated that quercetin reduced lactate production in human corneal stromal cells from keratoconus patients to levels comparable with healthy controls. This suggests a shift toward more efficient ATP production via the citric acid cycle. These findings support the hypothesis that quercetin may be effective in mitigating oxidative stress in keratoconus by modulating cellular energy metabolism [24,29,31].

In conclusion, quercetin is a highly promising flavonoid with significant therapeutic potential, exhibiting beneficial effects on the ocular surface through its antioxidant and anti-inflammatory mechanisms, validated in both in vitro and in vivo studies [24].

## 3. Dry Eye Syndrome and Eye Fatigue

Dry eye syndrome (DES) is also referred to as dry eye disease (DED) or Keratoconjunctivitis Sicca (KCS). It is a multifactorial ocular surface disorder characterized by discomfort and visual disturbances. According to the Tear Film and Ocular Surface Society (TFOS) Dry Eye Workshop II (DEWS II), dry eye is defined as follows: “A multifactorial disease of the ocular surface characterized by a loss of homeostasis of the tear film, and accompanied by ocular symptoms, in which tear film instability and hyperosmolarity, ocular surface inflammation and damage, and neurosensory abnormalities play etiologic roles.” [32].

Dry eye is traditionally classified into two main categories: aqueous-deficient and evaporative types [33]. However, these categories often overlap, and the disease is increasingly understood as a dynamic continuum rather than a binary classification. Recently, DES has gained recognition as a significant public health concern. The most frequent manifestations include burning, irritation, dryness, tearing, foreign body sensation, discomfort, and blurred vision. These symptoms can be persistent, significantly reducing the quality of life and posing a considerable challenge for both patients and clinicians.

Management of DES often depends on the underlying etiology, and monotherapy is frequently insufficient. For this reason, supportive therapies, including those based on plant-derived bioactive compounds, are being increasingly explored as adjunctive treatments. It is also important to note that while cataract remains the leading cause of blindness globally, dry eye disease is one of the most common postoperative complications following cataract surgery, even in cases without surgical complications [34].

### 3.1. Tea Tree Oil

TTO mentioned in the previous section has also demonstrated beneficial effects in the management of dry eye syndrome (DES). In a study by Mohammadpour et al. [34], significant improvements were observed in tear film parameters such as Tear Break-Up Time (TBUT), Ocular Surface Disease Index (OSDI) score, tear osmolarity, Uncorrected Distance Visual Acuity (UCDVA), and Corrected Distance Visual Acuity (CDVA) in both the treatment and control groups. However, the treatment group, which received eyelid scrubs containing TTO, showed statistically superior results in OSDI scores, TBUT, and osmolarity (*p* < 0.05). These findings underscore the efficacy of TTO-containing eyelid hygiene products in managing postoperative dry eye following cataract surgery [34].

Dry eye disease (DED) is one of the most common complications following photorefractive keratectomy (PRK), which is one of the methods of laser refractive surgery. However, the available epidemiological data on the incidence of dry eye syndrome after PRK are limited. The 2007 TFOS DEWS report indicated that in the case of LASIK surgery, the incidence of DED symptoms ranges widely from 0.25% to 48%, even in patients without previous complaints [35].

In a retrospective historical cohort study by Hasani et al. [36], the effectiveness of TTO in treating DES after PRK surgery was assessed. The study involved 64 patients experiencing dry eye symptoms post-PRK. The findings revealed greater symptom improvement in those who used tea tree oil-based shampoo, further supporting the therapeutic role of TTO in managing post-refractive surgery dry eye [37].

### 3.2. Manuka Honey

Manuka honey is a type of monofloral honey derived from the nectar of the Manuka tree’s blossoms. It is produced by honeybees (*Apis mellifera*) that collect nectar from the flowers of the *Leptospermum scoparium* plant, which is native to New Zealand. This honey contains a variety of components, including carbohydrates, minerals, proteins, fatty acids, phenolic compounds, and flavonoids [38]. Manuka honey has been shown to have anti-inflammatory and antioxidant effects, making it a potential treatment for dry eye syndrome. In meta-analysis, changes in subjective symptoms, tear film quality, ocular surface characteristics, adverse events, and patient compliance were examined over the longest follow-up period compared to baseline. The analysis included data from five randomized controlled trials involving a total of 288 adults with dry eye syndrome. The results revealed that treatment with Manuka honey led to significant improvements in several key measures, including the Ocular Surface Disease Index (OSDI) [39], Standard Patient Evaluation of Eye Dryness (SPEED) [40], tear evaporation rate, negative conversion rate of matrix metalloproteinase-9 (MMP-9) levels, ocular surface staining, and the frequency of daily lubricant use. Notably, no serious adverse events were reported, though some participants experienced temporary stinging and redness, which were generally well-tolerated [41].

### 3.3. Curcuma longa

*Curcuma longa* is an Indian spice turmeric that belongs to *Zingiberacea* family and is rich in curcuminoids, one of which is curcumin (diferuloylmethane). Curcumin was found to have anti-inflammatory, antioxidant, and antitumor properties, but due to its low solubility and oral bioavailability, it is not easy to exploit its full potential [42]. Curcumin has an ability to downregulate the expression of the IκBα gene, cyclooxygenase-2 gene (COX-2), prostaglandin E-2 (PGE-2), interleukin-1–6–8 (IL-1, IL-6, IL-8), and tumor necrosis factor-α (TNF-α) in synovial fibroblasts obtained from patients with rheumatoid arthritis. Additionally, it inhibits production of the reactive oxygen species, which reveals its antioxidant quality [43]. As in dry eye syndrome, there is an overproduction of IL-1β, IL-6, and TNF-α, as well as powerful activation of p38 MAP kinase, JNK MAP kinase, and NF-κB in corneal epithelial cells, the pretreatment with 5 mmol of curcumin significantly inhibited the previously mentioned activities [44].

### 3.4. Linum usitatissimum

*Linum usitatissimum* is a small plant from the *Linaceae* family and is commonly called flax. Its initial use was either for fiber or oil, but nowadays, it serves multiple purposes in various economy sectors [45]. As it contains vital fatty acids such as γ-linoleic acid (omega 6) and α-linoleic acid (omega 3), it was found to significantly reduce inflammation in patients with dry eye syndrome due to Sjögrens disease. Therapy was conducted with oral flaxseed oil capsules taken daily (1 or 2 g/day) [20].

### 3.5. Ginkgo biloba

Extracts of *Ginkgo biloba*, rich in flavonoids and terpenoids, have been shown to reduce oxidative stress and improve cellular resilience on the ocular surface. Clinical evidence indicates that topical formulations combining hyaluronic acid with *Ginkgo extract* can improve tear film stability and reduce postoperative dry eye symptoms, including conjunctival hyperemia and patient-reported discomfort [21]. Furthermore, experimental studies suggest that this extract may activate the Nrf2-mediated antioxidant defense pathway in human retinal pigment epithelial cells, indicating broader cytoprotective potential relevant to ocular surface health [22].

## 4. Cataract

Cataract remains the leading cause of blindness worldwide, accounting for approximately 47.9% of all cases and affecting an estimated 17 million individuals globally [23]. Among the various degenerative ocular disorders associated with aging, cataract is the most prevalent age-related complication. It is characterized by the progressive opacification of the crystalline lens, primarily attributed to cellular and molecular changes induced by aging, making age the most significant risk factor for cataract development.

Additionally, cataractogenesis often arises as an early secondary complication in individuals with diabetes mellitus, further increasing the global burden. The human lens, being a metabolically isolated and avascular structure with limited regenerative capacity, is particularly vulnerable to cumulative oxidative damage over time. Contemporary research supports the view that cataract formation is multifactorial in origin, involving genetic, metabolic, and environmental contributors [24].

At present, surgical extraction of the lens remains the only effective treatment for cataract, and it has become the most commonly performed surgical procedure among individuals over the age of 65. Nevertheless, given the growing demand and associated economic burden, there is a pressing need to develop accessible and cost-effective non-surgical therapeutic alternatives. Epidemiological projections indicate that delaying the onset of cataract by as much as ten years, regardless of the type of intervention, could reduce the number of cataract surgeries by up to 50%. [24,25,26]. At the molecular level, cataract formation has been closely linked to an elevated production of reactive oxygen species (ROS) and a depletion of endogenous antioxidants. ROS plays a pivotal role in protein aggregation, lipid peroxidation, and structural damage to lens fibers, ultimately contributing to lens opacification [27].

### 4.1. Quercetin

Quercetin, a dietary flavonol and the most abundant flavonoid consumed by humans, has shown promising protective effects against lens opacification, a hallmark feature of cataract formation [24,28,29]. The ocular lens is a metabolically secluded structure with limited regenerative capacity, making it especially vulnerable to cumulative oxidative damage over time. In age-related cataract, oxidative stress plays a central pathogenic role. In the setting of diabetes mellitus, cataract development is further exacerbated by glucose toxicity, nonenzymatic glycation, activation of the polyol pathway, and increased oxidative stress, all of which contribute to accelerated lens damage and opacification [23,31,32,33,34,35]

The potential of quercetin and related dietary polyphenols as chemoprotective agents in cataract prevention include the regulation of oxidative stress, inhibition of nonenzymatic glycation, modulation of the polyol pathway, suppression of calpain protease activity, and influence on lens epithelial cell signaling [24].

Moreover, the article addresses the important issue of quercetin bioavailability within the ocular lens, which continues to pose a significant challenge in translating in vitro findings into clinical practice. In particular, the antioxidant activity of flavonoids, considered the best-characterized biological property of this compound class, has been widely discussed in the scientific literature [24,37,38,41,42,43,44,45,46,47,48].

Flavonoids in general, and quercetin in particular, have been found to modulate specific signaling pathways in lens epithelial cells that are implicated in cataract progression. For instance, quercetin at a concentration of 0.1 mM has been shown to protect cultured human lens epithelial cells against the cytotoxic effects induced by 1% dimethyl sulfoxide (DMSO), further highlighting its potential therapeutic role [24,49,50,51,52,53].

In summary, an increasing body of experimental and epidemiological evidence supports the hypothesis that flavonoids, particularly quercetin, may play a role in cataract prevention, primarily through their antioxidant, anti-glycation, and signaling-modulatory properties [24].

### 4.2. Turmeric (Curcuma longa)

Curcumin, a polyphenolic compound derived from *Curcuma longa*, has attracted considerable attention for its potent antioxidant properties. A substantial body of research has indicated that curcumin may contribute to the prevention of cataracts by protecting the lens from oxidative damage, preserving antioxidant enzyme activity, and maintaining the balance of essential biomolecules involved in cellular defense mechanisms [54].

A significant mechanism through which curcumin exerts its anti-cataract effects is through the inhibition of oxidative stress-response proteins. In an in vitro study, Chhunchha et al. [55] demonstrated that curcumin effectively suppressed peroxiredoxin 6, a protein involved in cellular stress responses, in cultured human lens epithelial cells (hLECs). Oxidative stress has been identified as a pivotal factor in the development of lens opacification, thus the capacity of curcumin to modulate stress-related proteins suggests a potential protective role in the prevention of cataract onset [55].

Furthermore, curcumin has been demonstrated to enhance the activity of key antioxidant enzymes, which play a crucial role in neutralizing free radicals and protecting lens proteins from oxidative damage. In a study by Padmaja and Raju, it was observed that pretreatment with curcumin resulted in a substantial increase in the activity of superoxide dismutase (SOD) and catalase in Wistar rats. Since these enzymes help counteract oxidative stress, their upregulation suggests that curcumin strengthens the lens’s natural defense system, potentially slowing the development of cataracts [56].

A significant finding was the observation that curcumin preserves GSH levels, which is one of the most important protective mechanisms of curcumin. GSH, a potent antioxidant, plays a crucial role in protecting lens cells from lipid peroxidation and protein oxidation, both of which are hallmarks of cataract formation. In a study by Manikandan et al., it was observed that selenite exposure in rat lenses resulted in elevated levels of lipid peroxidation, leading to a substantial reduction in GSH levels. However, the administration of curcumin effectively restored GSH concentrations in both lens tissues and serum, thus confirming its role in mitigating oxidative damage [57,58].

### 4.3. Abrus precatorius

*Abrus precatorius* (commonly named the Rosary pea) is a plant that belongs to the *Fabaceae* family. Its natural habitat spreads across South Africa and southern part of Asia. Due to its abundance of chemically active compounds, such as amino acids and methyl ester, *A. precatorius* exhibits antimicrobial, anti-inflammatory, and antioxidant activity. That is the reason it is widely used in medicine [59]. It was proven in the in vitro examination of goat lenses that ethanolic extract of *A. precatorius* increases the amount of lipid hydroperoxides malondialdehyde and decreases the protein content, as well as stimulating Cu^2+^-induced lipoprotein diene formation and enzymatic and nonenzymatic antioxidants [60]. These results suggest that *A. precatorius* delays the progression of cataract, which pathogenesis is mostly based on oxidative damage (in this case, a calcium-induced one) [61].

### 4.4. Heliotropium indicum

*Heliotropium indicum* belongs to the *Boraginaceae* family and is also called an Indian heliotrope. However, it grows in other parts of the world (for example Africa) [62]. It was found to contain a lot of active alkaloids such as indicine, acetyl-indicine, indicinine-N-oxide, heleurine, heliotrine, supine, supinidine, and lindelofidine. There were two studies on rabbits’ eyes conducted by the same authors. It was proven that an extract of this plant has a significant hypotensive effect as well as antioxidant, neuroprotective, and anti-inflammatory properties. The other study reported a notable delay in cataractogenesis induced by galactose (the study was conducted on rats) [20].

## 5. Glaucoma

Glaucoma refers to a heterogeneous group of disorders with multifactorial etiologies, all sharing a common hallmark: progressive optic neuropathy characterized by structural damage to the optic nerve, apoptosis of retinal ganglion cells (RGCs), and corresponding visual field deficits. Visual impairment occurs as the degeneration of the optic nerve interrupts the transmission of visual information to the brain. Primary glaucoma is typically categorized into two major forms: open-angle glaucoma (OAG) and angle-closure glaucoma (ACG).

On a global scale, glaucoma represents the second leading cause of irreversible blindness, posing a significant and escalating public health challenge, particularly within the context of aging populations [63,64]. According to estimates from the World Health Organization, approximately 105 million individuals are affected by glaucoma worldwide, with an estimated 5 million people experiencing vision loss due to the disease [64].

Accurate diagnosis of glaucoma requires more than the measurement of intraocular pressure (IOP). A comprehensive assessment of the optic nerve head, the retinal nerve fiber layer, and the visual fields is essential. Parameters such as the vertical cup-to-disk ratio, particularly when evaluated in relation to optic disk size, represent critical indicators for early detection and effective monitoring [64].

Because glaucomatous optic neuropathy is irreversible, early diagnosis and prompt treatment are vital to preserving visual function [64]. While lowering IOP remains the cornerstone of glaucoma therapy, clinical evidence suggests that RGC loss may continue despite achieving target IOP levels in some patients [63]. This underscores the limitations of IOP-centric management strategies and points to the need for adjunctive neuroprotective approaches.

Neuroprotection in the context of glaucoma involves therapeutic interventions designed to prevent or delay the degeneration of retinal ganglion cells, independent of IOP control [64,65]. Both natural and synthetic compounds have been investigated for their neuroprotective effects. These include antioxidants, N-methyl-D-aspartate (NMDA) receptor antagonists, glutamate release inhibitors, calcium channel blockers, polyamine antagonists, and nitric oxide synthase inhibitors. Furthermore, cannabinoids, aspirin, melatonin, and vitamin B12 have also shown potential neuroprotective activity in preclinical and clinical studies [66].

### 5.1. Citicoline

Among the various agents currently under investigation, citicoline (cytidine-5′-diphosphocholine) has garnered particular interest due to its neuroprotective properties in glaucoma management [67,68]. Citicoline is a naturally occurring nucleotide compound composed of ribose, cytosine, pyrophosphate, and choline, and it functions as a biosynthetic precursor of phosphatidylcholine, which is a major phospholipid component of neuronal and mitochondrial membranes. It is water-soluble and exhibits excellent systemic bioavailability, exceeding 90%.

In recent years, citicoline has been increasingly used as an adjunctive treatment alongside standard intraocular pressure (IOP)-lowering therapies, particularly in patients with primary open-angle glaucoma (POAG). Both preclinical and clinical studies support its potential to provide long-term neuroprotection, primarily by reducing retinal ganglion cell (RGC) apoptosis, as well as short-term neuroenhancement through improvement of RGC function [68].

Multiple clinical studies have demonstrated improvements in visual field performance, visual evoked potentials, and pattern electroretinogram (PERG) readings following citicoline administration in glaucoma patients. Notably, recent findings indicate that oral supplementation with 500 mg of citicoline solution may significantly slow the progression of glaucomatous damage [25].

In a study conducted by Sahin et al. [69], the short-term effects of oral citicoline on retinal nerve fiber layer (RNFL) and macular ganglion cell–inner plexiform layer (mGCIPL) thickness in patients with POAG were evaluated. After three months of treatment, the citicoline group exhibited a significant increase in average RNFL thickness compared to baseline (*p* = 0.038). Moreover, increases in average and inferior quadrant RNFL thickness were significantly greater in the citicoline group compared to controls (*p* = 0.006 and *p* = 0.014, respectively). The authors concluded that oral citicoline therapy may effectively mitigate RNFL thinning in the short term, thereby reinforcing its role as a potential neuroprotective strategy in glaucoma management [69].

### 5.2. Cannabins

Since the 1970s, research has shown that both marijuana and its primary psychoactive component, tetrahydrocannabinol (THC), are capable of lowering intraocular pressure (IOP), which is a major risk factor in the pathogenesis of glaucoma [70]. Early findings generated considerable interest, especially because conventional glaucoma therapies of that era often caused significant adverse effects. Subsequent clinical studies confirmed that cannabinoids can reduce IOP as effectively as many standard antiglaucoma agents [71]. However, their short duration of action—typically only three to four hours—greatly limits therapeutic usefulness.

The precise mechanism by which cannabinoids reduce IOP remains unclear. Moreover, their clinical use is constrained by systemic hypotension, which may impair optic nerve perfusion, and psychoactive effects that are poorly tolerated by elderly patients, who represent the majority of glaucoma cases [70,71].

Nevertheless, a study by Bergman et al. explored glaucoma specialists’ perceptions Nevertheless, a survey by Bergman et al. indicated that over 25% of AGS members believe medical marijuana may have some potential role in glaucoma management, highlighting ongoing debate and interest in safer cannabinoid-based alternatives [72]. Reflecting these concerns, the American Glaucoma Society (AGS) has stated that medical marijuana is not an acceptable treatment option for glaucoma, emphasizing its short duration of action and undesirable systemic effects.

### 5.3. Saffron (Crocus sativus)

Saffron, derived from the pistils of *Crocus sativus* (*Iridaceae* family), is rich in carotenoid derivatives such as crocin and crocetin, which possess strong antioxidant and radical-scavenging properties [73]. Historically employed in traditional Asian and Persian medicinal practices, particularly for addressing depressive symptoms [74], saffron has also demonstrated potential benefits for ocular health [75].

Recent research highlights the potential benefits of saffron in glaucoma management, both in human and animal studies. A pilot study conducted on patients with POAG demonstrated that oral aqueous saffron extract contributed to a reduction in IOP in individuals already receiving timolol and dorzolamide therapy. This effect became noticeable after three weeks of supplementation, reaching its peak by the fourth week. However, following a washout period, IOP gradually returned to baseline within a month after discontinuation. The observed ocular hypotensive effect of saffron is likely linked to its rich composition of bioactive carotenoid derivatives, primarily crocin, and crocetin, known for their potent antioxidant and neuroprotective properties [76].

In addition to clinical findings, experimental studies in a glaucoma mouse model have provided further evidence of saffron’s therapeutic potential. Research has confirmed that saffron extract exhibits neuroprotective and anti-inflammatory effects, playing a crucial role in mitigating glaucoma-related damage. Specifically, saffron treatment led to a reduction in microglial cell count and suppressed microglial activation in both hypertensive (OHT) and normotensive eyes. Given that microglial overactivation is associated with neuroinflammation and retinal ganglion cell degeneration in glaucoma, these results suggest that saffron may exert protective effects by modulating inflammatory pathways. One proposed mechanism involves the regulation of P2RY12 expression, a key receptor implicated in microglial activity and immune response regulation [77].

### 5.4. Foeniculum vulgare

*Foeniculum vulgare*, commonly named fennel, is a seasonal herb that grows in the wild and is cultivated in fields as well. It is an inseparable ingredient in some European cooking. However, it also has a medical value [78]. Fruit and root have relaxant, estrogenic, analgesic, and anti-inflammatory properties. Meanwhile, seeds have been shown to have estrogenic, antioxidant, and antihirsutism activities; it increases milk secretion, promotes menstruation, facilitates birth, alleviates the symptoms of dysmenorrhea, and increases libido, female climacteric, as well as the antifungal property of essential oil [79]. Glaucoma major risk factor is an elevated IOP, which was proven to be lowered by *F. vulgare* seed extract in the research conducted on rabbits. It was suggested that the given effect is due to *F. vulgare* anticholinesterase activity [80].

### 5.5. Ginkgo biloba L.

*Ginkgo biloba* L. is one of the most popular herbal supplements used around the world. These trees are called “living fossils” and their main chemical compounds are terpenoids, flavonoids, and proanthocyanides. Its role in glaucoma treatment relies on increasing the ocular blood flow, which helps to protect retinal ganglion cells [81]. *G. biloba* L. was found to be useful as an adjuvant in glaucoma resistant to other treatments [82]. Its antioxidant, anti-inflammatory and vasoregulatory properties are used in glaucoma treatment. It stabilizes mitochondria to prevent oxidative stress, reduces active cells in low-grade inflammation, and has a vasodilatory action. Randomized clinical trials have proven that *G. biloba* L. significantly increases ocular blood flow, blood volume, and velocity. Another study reported changes in superior and inferior retinal nerve fiber layer thickness, malondialdehyde (a plasma-derived oxidative stress marker), and glutathione peroxidase (an antioxidant enzyme) in patients with primary open-angle glaucoma (POAG), but it found no significant changes in IOP. Both studies noted improvements in visual fields [83].

### 5.6. Physostigma venenosum

*Physostigma venenosum* can be found in tropical forests of Africa. Its seed are known as Calabar beans, and they are the part of plant with the most medical value [84]. The first herbal medicine for glaucoma was isolated from these seeds—an alkaloid called physostigmine. Physostigmine is a common acetylcholinesterase inhibitor, which leads to miosis, causing the circular portion of the ciliary muscle to contract in order to accommodate. This action reduces IOP. This is due to multiple side effects, including headache, blurred vision, and an increased risk of inflammation or retinal detachment [20].

### 5.7. Pilocarpus jaborandi

*Pilocarpus jaborandi* is another plant from which a natural miotic drug pilocarpine was isolated. *P. jaborandi* derives from subtropical regions of Brazil. Pilocarpine acts directly on muscarinic receptors of the pupil sphincter and on the ciliary muscle, which lead to reduction in IOP. It has more tolerable side effects than physostigmine and is still used to this day [20].

## 6. Age-Related Macular Degeneration (AMD)

Age-related macular degeneration (AMD) is a progressive retinal disease that results in the gradual loss of central vision due to degeneration of the macular region [85]. The advancement of AMD is associated with characteristic histopathological alterations, including pigmentary disturbances, drusen formation, thickening of Bruch’s membrane, and basal laminar deposits. Drusen, which consist of lipid-rich material deposited beneath the retinal pigment epithelium (RPE), are clinically visible as pale-yellow spots on the retina [86].

With advancing age, the risk of oxidative damage to cellular components increases, leading to dysfunction and tissue degeneration. Both the RPE and the macular retina are highly susceptible to oxidative stress. Additional lifestyle-related risk factors, such as cigarette smoking, alcohol consumption, and poor dietary habits, further contribute to AMD development [87]. Current therapeutic strategies for wet AMD primarily aim to reduce oxidative stress through antioxidants and to limit pathological neovascularization by inhibiting vascular endothelial growth factor (VEGF) release and expression with anti-VEGF compounds [88].

In parallel, increasing scientific attention has focused on the role of plant-derived bioactive compounds in AMD prevention and treatment. Carotenoids such as lutein, zeaxanthin, and crocin, together with polyphenols, represent the most frequently studied natural agents, and their synergistic effects have been associated with improved outcomes. Among medicinal plants, saffron, ginkgo biloba, bilberry and blueberry, and turmeric (curcuma) are the most widely reported for their beneficial properties [87]. Curcuma and ginkgo exhibit both antioxidant and anti-angiogenic activities, highlighting their potential therapeutic relevance in AMD management.

### 6.1. Saffron

Over the past decade, growing evidence has emphasized the pharmacological properties of saffron and its bioactive constituents. Notably, saffron has attracted attention for its neuroprotective potential, including in the context of age-related macular degeneration (AMD). Clinical trials have provided essential evidence supporting the neuroprotective role of saffron supplementation. Among medicinal plants, saffron is of particular interest because it can improve visual function at doses considered safe and well below toxic thresholds [89,90,91,92,93].

The antioxidant activity of saffron is largely attributed to its carotenoid compounds, such as crocin and crocetin [94,95]. Both short-term trials and longer-term follow-ups have reported improvements in visual function following saffron supplementation. However, a direct quantitative comparison of study outcomes is challenging due to variations in formulation, dosage, treatment duration, test methodologies, and outcome measures across studies.

From a toxicological standpoint, saffron is considered safe for human consumption. Clinical data indicate that a daily dose of 30 mg is both efficacious and well-tolerated, while toxic effects have been reported only at doses of 5 g or higher. The lethal dose is estimated to be approximately 20 g [89,94,95].

### 6.2. Bilberry (Vaccinium myrtillus)

Bilberries native to the northern regions of Europe are considered valuable natural resources due to their high concentration of phenolic compounds [96]. Their abundance in the northern European landscape has led to their widespread use in traditional medicine and modern dietary supplements, owing to their potential health benefits, including their role in protecting against oxidative stress and inflammation [97].

Pigmented rabbits, selected for their melanin-rich retinal pigment epithelium (RPE) that resembles the human retina, were used to develop a model of visible light-induced retinal damage. The animals were exposed to 18,000 lx light for a period of two hours and then sacrificed on day 7 in order to assess the protective effects of bilberry anthocyanin extract (BAE) at doses of 250 and 500 mg/kg/day. The results demonstrated that BAE treatment significantly preserved retinal function by maintaining the thickness of the outer nuclear layer and photoreceptor outer segment length while inhibiting apoptotic markers (Bax, Bcl-2, and caspase-3). Furthermore, it enhanced antioxidant defenses (superoxide dismutase, glutathione peroxidase, and catalase), reduced lipid peroxidation, and suppressed proinflammatory cytokines (IL-1β) and angiogenic factors (VEGF). Bilberry (*Vaccinium myrtillus*), which is abundant in anthocyanins, has been shown to possess antioxidative, anti-inflammatory, and anti-apoptotic properties that help neutralize reactive oxygen species (ROS) and regulate cellular pathways, potentially delaying the progression of age-related macular degeneration (AMD). In addition, anthocyanins have been demonstrated to facilitate rhodopsin regeneration and enhance visual function [98].

### 6.3. Spinacia oleracea, Brassica oleracea, Capsicum annuum as Major Sources of Xantophylls

Xantophylls are carotenoids that consist of carbon and hydrogen as well as hydroxyl groups, which make them more hydrophilic than carotenes (the other group of carotenoids that is made only of carbon and hydrogen). Xanthophylls (lutein and zeaxanthin) accumulate in the eye and are responsible for protecting retina from harmful photochemical reactions (leading to, for example, age-related macular degeneration (AMD)) due to their antioxidant and anti-inflammatory properties. Another compound of this group—β-Cryptoxanthin—can be partially metabolized to vitamin A. As xanthophylls are not synthesized de novo within human body, they can be only obtained in diet [99]. The major sources of xanthophylls are *Spinacia oleracea*, *Brassica oleracea*, and *Capsicum annuum* (accordingly spinach, kale and pepper). A clinical trial AREDS1 (supplementation of β-carotene, vitamin C, vitamin E, zinc, and copper) conducted by The National Eye Institute lowered progression to advanced AMD by 25%. Adding lutein and zeaxanthin to these supplements lowered the progression by another 10%. Additionally, replacing 15 mg β-carotene (it was found to increase a risk of lung cancer in smokers) with 10 mg lutein and 2 mg zeaxanthin in the AREDS1 study resulted in progression lowered by 22% [100].

### 6.4. Scutellaria baicalensis

*Scutellaria baicalensis* from the *Lamiaceae* family is vastly distributed in China, Russia, Mongolia, North Korea, and Japan and is still used in Chinese medicine [101]. It is rich in bioactive flavonoids such as baicalein (5,6,7-trihydroxyflavone), baicalin (5,6-dihydroxy-7-O-glucuronide), and wogonin (5,7-dihydroxy-8-methoxyflavone). These compounds have been proven to have anti-inflammatory, antioxidant, and anti-angiogenic effects on ocular tissues. Their antilipoxygenase activity is supposed to take part in reducing inflammation in AMD. Moreover, they exhibit protective properties on several layers of retina [102].

### 6.5. Quercetin

Quercetin’s strong antioxidant and anti-inflammatory effects help counteract oxidative damage to photoreceptors and retinal pigment epithelium (RPE), which is central to AMD pathology. Experimental studies show that quercetin reduces reactive oxygen species, suppresses endoplasmic reticulum stress pathways, and inhibits apoptosis in photoreceptor cells exposed to toxic retinal byproducts such as all-trans-retinal. Additionally, quercetin modulates senescence-related signaling and enhances cellular defense mechanisms, supporting the integrity of aging retinal tissue [103]. While evidence remains largely preclinical, these findings suggest that quercetin may offer meaningful protection against the oxidative and degenerative processes underlying AMD.

## 7. Diabetic Retinopathy

Diabetic retinopathy (DR) is a microvascular complication of diabetes that affects the eyes and is one of the leading causes of vision impairment and blindness among diabetic patients. It is estimated that up to 80% of individuals who have lived with diabetes for 20 years or more will develop some form of DR. Importantly, over 90% of new cases could be prevented or significantly delayed with regular eye monitoring and appropriate treatment. The duration of diabetes is a major risk factor for diabetic retinopathy (DR). The longer an individual has diabetes, the higher the likelihood of developing this complication. These statistics underscore the critical need for preventive strategies and effective therapies. In recent years, there has been growing interest in natural products for pharmacological intervention, largely due to the greater structural diversity and biological compatibility of phytochemicals compared to synthetic compounds. Emerging research has explored the potential of several conventional medicinal herbs in preventing and managing diabetic retinopathy. Notably, herbs such as *Azadirachta indica*, *Ginkgo biloba*, *Anisodus tanguticus*, *Pinus pinaster*, *Salvia miltiorrhiza*, *Stephania tetrandra*, and *Gymnema sylvestre* have shown promising therapeutic effects. This growing body of evidence is supported by a range of epidemiological, in vitro, and in vivo studies, which suggest that these natural compounds may interrupt key pathogenic pathways involved in DR, including oxidative stress, inflammation, and vascular dysfunction [104].

### 7.1. Carrot (Daucus carota)

The carrot is a root crop that is cultivated on a wide scale. It is a member of the *Apiaceae* family and is grown annually for the consumption of its edible roots. It flourishes in tropical and subtropical regions from September to November, while temperate climates permit year-round cultivation [105]. The seeds of the carrot require cool temperatures for their production, and the root itself is a rich source of carotenoids and flavonoids, which contribute to its antioxidant properties and diverse pigmentation. Carrots are a rich source of beta-carotene, a precursor of vitamin A, which is essential for optimal vision [106].

The objective of the study conducted by El-Mansi et al. [107] was to ascertain the detrimental effects of diabetes on retinal visual functions and to determine the potential of supplementation with vitamin A and carrot root extract (CE) to safeguard the retina in rats with hyperglycemia. The experimental model included 50 male Wistar albino rats. The results showed that diabetic rats had a significant reduction in retinal thickness, an increased number of apoptotic ganglion cells, and degeneration of the synaptic layers compared with control rats. The inner retina displayed increased neovascularization, while the outer retina exhibited vacuolar degeneration of the photoreceptor cell layer. Biochemical analyses revealed a decrease in antioxidant enzyme levels (CAT, SOD, and GST) and an increase in lipid peroxidation. Furthermore, cellular angiogenic and stress markers exhibited significant elevation, accompanied by augmented apoptotic activity, as indicated by increased annexin-V and PARP expression. Furthermore, alterations in retinal neurotransmitter levels were observed in diabetic rats in comparison to both the control and treated groups. The supplementation of vitamin A and CE proved effective in alleviating these retinal impairments in diabetic rats, restoring the assessed parameters to near-normal levels. The protective effect of both treatments is suggested by their ability to reduce oxidative stress, prevent neuronal degeneration, and preserve visual functions [107].

### 7.2. Zingiber officinale

*Zingiber officinale*, commonly known as ginger, belongs to the *Zingiberaceae* family. It has been used by Ayurvedic and Chinese medicine to treat cardiovascular and gastrointestinal problems as well as cold, lungs diseases, and arthritis. Ginger suppresses prostaglandin synthesis through inhibition of cyclooxygenase-1 and cyclooxygenase-2. Much research has proven that the ginger extract also has antimicrobial activity and antioxidant properties [108]. The main active component is gingerol. In a study conducted on rats using an extract containing 5% of 6-gingerol, the results indicated a significant reduction in hyperglycemia, the diameter of the retinal vessels, and vascular basement membrane thickness. Structural changes in retinal vasculature were linked to lowered expression of nuclear factor-κB (NF-κB) and reduced activity of tumor necrosis factor-α (TNF-α) and vascular endothelial growth factor (VEGF) in the retinal tissue [19].

### 7.3. Perilla frutescens

*Perilla frutescens* is an herb from *Lamiaceae* family and is mostly cultivated in China, Japan, India, Thailand, and Korea. It was found to contain phenolic compounds such as rosmarinic acid, caffeic acid, and ferulic acid as well as flavonoids (like lutein) [109]. These phenolic compounds demonstrate aldose reductase (AR)-inhibitory activity. It stops the excessive conversion of glucose to sorbitol, which accumulates in various organs causing diabetic complications (like diabetic retinopathy). Luteolin, apigenin, and diosmetin, isolated from *P. frutescens* seeds, also showed AR inhibitory activity. The strongest AR inhibitors were found to be lutein and rosmarinic acid (accordingly IC_50_ of 1.89 µM and IC_50_ of 2.77 µM). These compounds also exhibit α-glucosidase-inhibitory activity. α-glucosidase is an enzyme that breaks down α (1–4) bonds in oligo- and disaccharides to come into being simple sugars. This activity has been successfully used to treat type II diabetes. The most hope is put on lutein with an IC_50_ value of 45.4 µM. This study suggests *Perilla frutescens* could prevent complications developing from diabetes [110].

### 7.4. Quercetin

In diabetic retinopathy, quercetin demonstrates notable neuroprotective, anti-inflammatory, and anti-angiogenic activities, positioning it as a promising candidate for mitigating early retinal injury associated with diabetes. Preclinical studies using streptozotocin-induced diabetic rat models have shown that quercetin effectively attenuates retinal oxidative stress, downregulates the expression of pro-angiogenic and matrix-remodeling factors such as VEGF and MMP-9, and preserves overall retinal architecture. Furthermore, quercetin has been reported to reduce neuronal apoptosis through modulation of mitochondrial signaling pathways, including suppression of cytochrome c release and caspase-3 activation, while concurrently enhancing levels of neurotrophic factors such as BDNF [111,112]. Collectively, these mechanisms contribute to the stabilization of the retinal microenvironment and curb the neurovascular dysfunction that typifies diabetic retinopathy.

## 8. Critical Summary of Plant Extracts in Eye Diseases

Despite the growing interest in plant-based therapies for ocular disorders (Table 1), the current body of evidence remains limited and fragmented.

From nearly 3000 articles screened in meta-analysis, only seven studies met the inclusion criteria, and these were of low-to-moderate quality. The small number of eligible studies highlights a major gap in reliable data. A central concern is the heterogeneity of available research. Observational and experimental designs varied widely in methods, outcome measures, and reporting standards, producing inconsistency that reduces confidence in the pooled results. High statistical heterogeneity (I^2^ values above 85%) and clear signs of publication bias further weaken the conclusions. Another important limitation is the scarcity of clinical evidence. Much of the available information comes from ethnobotanical surveys or experimental studies in animals. While these provide useful insights, they cannot substitute for well-designed clinical trials. Consequently, the actual therapeutic value and safety profile of many plants used in eye care remain uncertain [123].

The lack of standardization of plant preparations represents an additional risk. Variability in species, growing conditions, and extraction methods lead to differences in the concentration of active compounds. Without standardized formulations, reproducibility and safety cannot be guaranteed [124,125].

Moreover, documented clinical reports from Central Saudi Arabia illustrate the severe ocular complications that may arise from the direct use of traditional herbal remedies. Substances such as kermes dye, alum, Calotropis (Ushaar) latex, honey, aloe, and *Lepidium sativum* have been associated with conjunctival scarring, symblepharon formation, keratinization, corneal thinning and ulceration, and even endophthalmitis. These harmful outcomes often result from unknown composition, contamination, or delayed presentation to medical care. Such evidence underscores the need for cautious evaluation of folkloric practices, public health education, and strict clinical vigilance when patients report the use of traditional eye medications [126].

## 9. Conclusions

Plants play an important role in ophthalmology, offering natural remedies for the treatment and prevention of eye diseases such as conjunctivitis, cataracts, glaucoma, and AMD. Many traditional herbal therapies show therapeutic potential, but their efficacy and safety require further clinical trials. Although phytomedicine is a valuable adjunct to conventional treatments for eye disease, its full implementation requires solid scientific evidence and further research into the mechanisms of action of individual compounds.

## Figures and Tables

**Table 1:** Summary of natural plant-derived compounds with potential ocular health benefits.

Plant/Compound	Active Substance(s)	Mechanisms of Action	Ocular Condition/Benefit	References
Flavonoids (general)	Various polyphenols	Antioxidant, anti-inflammatory	Reduction in oxidative stress in ocular tissues	[30,113,114,115,116,117,118,119]
Quercetin	Quercetin	Anti-inflammatory, antioxidant	Protection against oxidative stress and inflammation in ocular diseases	[23,24,28,29,31,32,33,34,35]
Tea tree oil (*Melaleuca alternifolia*)	Terpinen-4-ol and others	Antimicrobial, anti-inflammatory	*Demodex* blepharitis, ocular surface inflammation, dry eye relief	[12,13,15,36]
Curcumin (*Curcuma longa*)	Curcumin	Antioxidant, anti-inflammatory, anti-angiogenic	Potential protective role in retinal degeneration and oxidative eye disorders	[120,121,122]
Alkaloids	Berberine, pilocarpine, caffeine	Neuroprotective, cholinergic agonist (pilocarpine), antioxidant/vasomodulatory (berberine, caffeine)	Lowering IOP (pilocarpine), retinal protection, enhanced ocular blood flow	[18,19,20]
Ginger (*Zingiber officinale*)	Gingerol, shogaol	Anti-inflammatory, antioxidant	Reduction in ocular surface inflammation, potential protection against oxidative retinal injury	[108]
Saffron (*Crocus sativus*)	Crocin, safranal	Antioxidant, neuroprotective	Improvement in retinal function, benefits in AMD and glaucoma	[7,89,94,95]
Aloe vera	Polysaccharides	Anti-inflammatory, supports healing	Ocular surface protection, wound healing	[2,3,4]
Bilberry (*Vaccinium myrtillus*)	Anthocyanins	Antioxidant, vascular protection	Protective effect on retina	[5,96,97,98]
Carrot (*Daucus carota*)	β-carotene (Vitamin A)	Antioxidant, visual cycle support	Vision support, prevention of deficiency-related disorders	[105,106,107]

## Data Availability

No new data were created or analyzed in this study. Data sharing is not applicable to this article.
